# Butanol Isomers Exert Distinct Effects on Voltage-Gated Calcium Channel Currents and Thus Catecholamine Secretion in Adrenal Chromaffin Cells

**DOI:** 10.1371/journal.pone.0109203

**Published:** 2014-10-02

**Authors:** Sarah McDavid, Mary Beth Bauer, Rebecca L. Brindley, Mark L. Jewell, Kevin P. M. Currie

**Affiliations:** 1 Department of Anesthesiology, Vanderbilt University School of Medicine, Nashville, Tennessee, United States of America; 2 Department of Pharmacology, Vanderbilt University School of Medicine, Nashville, Tennessee, United States of America; 3 Department of Anesthesiology, Department of Pharmacology, and Vanderbilt Brain Institute, Vanderbilt University School of Medicine, Nashville, Tennessee, United States of America; University of Waterloo, Canada

## Abstract

Butanol (C_4_H_10_OH) has been used both to dissect the molecular targets of alcohols/general anesthetics and to implicate phospholipase D (PLD) signaling in a variety of cellular functions including neurotransmitter and hormone exocytosis. Like other primary alcohols, 1-butanol is a substrate for PLD and thereby disrupts formation of the intracellular signaling lipid phosphatidic acid. Because secondary and tertiary butanols do not undergo this transphosphatidylation, they have been used as controls for 1-butanol to implicate PLD signaling. Recently, selective pharmacological inhibitors of PLD have been developed and, in some cases, fail to block cellular functions previously ascribed to PLD using primary alcohols. For example, exocytosis of insulin and degranulation of mast cells are blocked by primary alcohols, but not by the PLD inhibitor FIPI. In this study we show that 1-butanol reduces catecholamine secretion from adrenal chromaffin cells to a much greater extent than tert-butanol, and that the PLD inhibitor VU0155056 has no effect. Using fluorescent imaging we show the effect of these drugs on depolarization-evoked calcium entry parallel those on secretion. Patch-clamp electrophysiology confirmed the peak amplitude of voltage-gated calcium channel currents (*I_Ca_*) is inhibited by 1-butanol, with little or no block by secondary or tert-butanol. Detailed comparison shows for the first time that the different butanol isomers exert distinct, and sometimes opposing, effects on the voltage-dependence and gating kinetics of *I_Ca_*. We discuss these data with regard to PLD signaling in cellular physiology and the molecular targets of general anesthetics.

## Introduction

Butanol (C_4_H_10_OH) exists as multiple isomers; primary (1-butanol) and secondary butanol (2-butanol) have a straight carbon chain with the hydroxyl group located at the terminal or second carbon, respectively. The four-carbon chain can also be branched with the hydroxyl group located at a terminal carbon (isobutanol; also called 2-methyl-1-propanol) or at the internal carbon (tert-butanol; also called 2-methyl-2-propanol). Like other primary alcohols, 1-butanol serves as a substrate for phospholipase D (PLD) [1]. In the absence of alcohols, PLD catalyzes the hydrolysis of phosphatidylcholine to produce choline and phosphatidic acid, with the latter acting directly or indirectly through intracellular signaling cascades to control a diverse array of cellular functions [2]–[11]. However, in the presence of 1-butanol, PLD produces phosphatidylbutanol rather than phosphatidic acid [1]. Neither secondary nor tertiary butanols undergo this transphosphatidylation, so these alcohols are commonly used as controls for 1-butanol to implicate phosphatidic acid/PLD signaling. Recently, several selective pharmacological inhibitors of PLD have been developed [12]–[14], and some reports have noted a disparity between the effects of these and 1-butanol on cellular function. For example, 1-butanol inhibits insulin secretion, but the PLD inhibitor FIPI has no effect [12]. Similarly, 1-butanol inhibits membrane ruffling, blebbing, and degranulation (exocytosis) in mast cells, but FIPI does not [15]. The implication from these studies is that acute application of 1-butanol blocks exocytosis by a mechanism(s) distinct from PLD signaling.

This raises the question by which other target(s) might butanol inhibit exocytosis? It is known that primary alcohols induce anesthesia and act in a manner similar to inhalational anesthetics [16]. Potential molecular targets for alcohols/general anesthetics include ligand-gated, voltage-gated, and other ion channels [17]–[23]. Although much attention has focused on post-synaptic ligand-gated ion channels, there is also evidence for presynaptic effects of alcohols/anesthetics [23]–[25]. The critical roles of voltage-gated channels make them an attractive potential target for disruption of stimulus-secretion coupling by butanol (and other alcohols/anesthetics). Voltage-gated sodium and potassium channels control electrical excitability, while calcium entry through voltage-gated calcium channels controls a multitude of cellular functions including vesicular trafficking and neurotransmitter/hormone secretion. Indeed, primary alcohols have been shown to modulate voltage-gated channels: 1-butanol inhibits voltage-gated sodium channel currents [26], voltage-gated potassium channel currents [27]–[29], and voltage-gated calcium channel currents (*I_Ca_*) [30]–[32]. However, it is not known if butanol isomers differ in their modulation of voltage-gated ion channel function.

In this study we investigated the effects of butanol isomers on catecholamine secretion and *I_Ca_* in adrenal chromaffin cells, an important neuroendocrine component of the sympathetic nervous system and a well-characterized neurosecretory model [33]. Both PLD1 and PLD2 are expressed in chromaffin cells, and PLD1 has been implicated in controlling catecholamine secretion [11], [34]. However, to our knowledge the effects of newer PLD inhibitors have not been reported prior to this study. We show that 1-butanol reduces catecholamine secretion to a much greater extent than tert-butanol, but VU0155056, a newly developed cell membrane permeable inhibitor of both PLD1 and PLD2 [13], [14], has no effect. Fluorescent imaging approaches reveal parallel effects on depolarization-evoked calcium entry. With patch-clamp electrophysiology we demonstrate concentration-dependent inhibition of *I_Ca_* by 1-butanol with little or no block by secondary or tert-butanol. Detailed comparison shows for the first time that different butanol isomers exert distinct, and sometimes opposing, effects on the gating and kinetics of *I_Ca_*. We discuss these data with regard to PLD signaling in cellular physiology and the molecular targets of general anesthetics.

## Materials and Methods

### Cell culture

Bovine adrenal glands were obtained from a local slaughterhouse (C & F Meat Co. Inc., College Grove, TN), and chromaffin cells were prepared by digestion with collagenase followed by density gradient centrifugation as described previously [35]. The cells were plated onto coverslips coated with collagen (at a density of 0.3–0.4×10^6^ cells/mL for [Ca^2+^]_i_ measurements or ∼0.2×10^6^ cell/mL for patch clamp recordings). For secretion studies, cells were plated in 24-well tissue culture plates at a density of ∼0.3×10^6^ cells per well. Fibroblasts were effectively suppressed with cytosine arabinoside (10 µM) (Sigma-Aldrich; St Louis MO), leaving relatively pure chromaffin cell cultures. The culture medium consisted of Dulbecco's modified Eagle medium \ F12 (1∶1) supplemented with fetal bovine serum (10%), glutamine (2 mM), penicillin/streptomycin (100 unit mL^−1^/100 µg mL^−1^), cytosine arabinoside (10 µM) and 5-fluorodeoxyuridine (10 µM). Tissue culture reagents were from Life Technologies (Grand Island, NY). The culture medium was replaced the day after isolation and experiments were performed 2–5 days following cell isolation.

### [Ca^2+^]_i_ Measurements

Free cytosolic Ca^2+^ concentration ([Ca^2+^]_i_) was measured using an InCyt IM2 fluorescence imaging system (Intracellular Imaging Inc., Cincinnati, OH) and a Nikon TE2000 fluorescence microscope (Nikon Instruments Inc., Melville, NY) [35]. Cells were washed twice with HEPES-buffered Hanks Balanced Salt Solution and incubated in the dark for 30–45 minutes with 3-5 µM FURA2-AM or FURA4F-AM at 37°C. Cells were then washed (in the dark) in FURA-free solution for 30–60 minutes before recording. The coverslip was transferred to a recording bath (volume ∼300–400 µL) and continually perfused with fresh solution at a flow rate of ∼4 ml/min from gravity-fed reservoirs. Cells were excited at 340 nm and 380 nm and emission at 510 nm detected using a Pixelfly digital camera. The ratio of fluorescence emission at 340 nm/380 nm excitation was collected every 1–2 s throughout the experiment and converted to [Ca^2+^]_i_ using an *in vitro* calibration curve. Data were transferred to OriginPro software (Originlab Corporation, Northampton, MA) for analyses.

### Catecholamine secretion experiments

Cells in 24-well plates were washed twice with extracellular solution and equilibrated in this solution for 30 mins at ∼37°C. This was next replaced with fresh solution to determine basal release or with solution containing 30 mM KCl to stimulate secretion. After a five-minute stimulation period at ∼37°C the cells were placed on ice, and the solution was removed and added to an equal volume of ice-cold 0.4 M perchloric acid. The cells were lyzed by addition of perchloric acid and scraping to extract the non-secreted catecholamines. The catecholamine content of the samples was determined by a specific high performance liquid chromatography (HPLC) assay utilizing an Antec Decade (oxidation potential: 0.7 V) electrochemical detector in the Neurochemistry Core of the Vanderbilt Brain Institute as described previously [35]. The amount of catecholamine secreted during the 5-minute stimulation period was expressed as a percentage of the total catecholamine content for that dish of cells. For each experiment, duplicate wells for each condition (control and drug treated) were averaged (to yield n = 1). 1-butanol (0.4% v/v; ∼44 mM), tert-butanol (0.4% v/v; ∼42 mM), the PLD inhibitor VU0155056 (1 µM), or DMSO (0.01%) (vehicle for VU0155056) was added to the cells for 10 minutes during the preincubation period and throughout the stimulation with KCl. Statistical analyses were performed using Prism5 software (GraphPad Software Inc., La Jolla, CA).

### Patch-clamp electrophysiology

Patch pipette electrodes were pulled from borosilicate glass capillary tubes (World Precision Instruments, Sarasota, FL) using a Sutter P-97 pipette puller (Sutter Intrument, Novato, CA), coated with dental wax (Electron Microscopy Sciences, Hatfield, PA) and fire-polished to a final resistance of ∼2 MΩ when filled with a CsCl-based internal solution. Cells were voltage-clamped in the conventional whole-cell configuration using either an Axopatch 200B amplifier, Digidata 1400A interface, and PClamp10 (Clampex) acquisition software (Molecular Devices, Sunnyvale, CA) or a HEKA EPC10 amplifier and PatchMaster acquisition software (HEKA instruments). Analog data were filtered at 2–3 kHz and digitized at 50 kHz. Series resistance was partially compensated (∼50–80%) and data for *I*_Ca_ were subjected to linear capacitance and leak subtraction using P/N protocols (P/-4 or P/-8) with the leak pulses applied following the test pulses. The recording bath (volume ∼300 µL) was continually perfused with fresh solution at a flow rate of ∼4 ml/min from gravity-fed reservoirs. The exception was when ω-conotoxin GVIA (CgTx) was used to block the N-type *I_Ca_*. For these experiments the CgTx was added as a 50 µL bolus of 2 µM CgTx into a static bath. The estimated final concentration of CgTx was ∼0.5–1 µM. Blockade of the channels by CgTx is irreversible over the time course of these experiments. Following plateau of the current inhibition by CgTx (approximately 4–5 minutes), the bath flow was restarted and butanol applied. Raw data were analyzed using PClamp10 (Clampfit) or HEKA FitMaster software. Graphing and statistical analyses were performed using Origin or Prism.

### Solutions, drugs and reagents

The extracellular solution for calcium imaging and secretion studies comprised (in mM): 136 NaCl, 2 KCl, 1 MgCl, 10 glucose, 10 HEPES, 2 CaCl, pH 7.3 osmolarity ∼305 mOsm. The extracellular solution for patch-clamp recording was (in mM): 136 NaCl, 2 KCl, 1 MgCl, 10 glucose, 10 HEPES, 5 CaCl, pH 7.3 osmolarity ∼310 mOsm. Tetrodotoxin (TTX) (0.5–1 µM) was added to block voltage-gated sodium channels. The intracellular (patch pipette) solution consisted of (in mM): CsCl 110, MgCl_2_ 4, HEPES 20, EGTA 10, GTP 0.35, adenosine triphosphate 4, creatine phosphate 14, pH 7.3, osmolarity ∼310–315 mOsm.

The AM-ester of FURA was prepared as 1 mM stock in dimethyl sulfoxide (DMSO) and aliquots frozen until use. The pentapotassium salt used for in vitro calibration curves was prepared as an aqueous stock and stored at 4°C. Butanol was added to the extracellular solutions at the indicated concentrations on the day of each experiment. VU0155056 was kindly provided by Dr. Alex Brown (Vanderbilt University) (also available from Avanti Polar Lipids Inc., Alabaster, Alabama). Stock solutions were prepared in DMSO (10 mM) and diluted into extracellular solution (1 µM final concentration) on the day of use. Tetrodotoxin (R&D systems, Minneapolis, MN) was prepared as a 1 mM aqueous stock and aliquots frozen until use (final concentration when diluted into extracellular solution was ∼0.5 µM). ω-conotoxin GVIA (R&D systems, Minneapolis, MN) was prepared as a 100–300 µM aqueous stock and stored as frozen aliquots until use. On the day of the use, the aliquot was diluted to 2 µM in extracellular solution and a 50 µL bolus of this solution was added to the static recording bath (300–400 µL).

## Results

### Differential inhibition of calcium entry and catecholamine secretion by butanol isomers and the PLD inhibitor VU0155056

**Figure 1:**
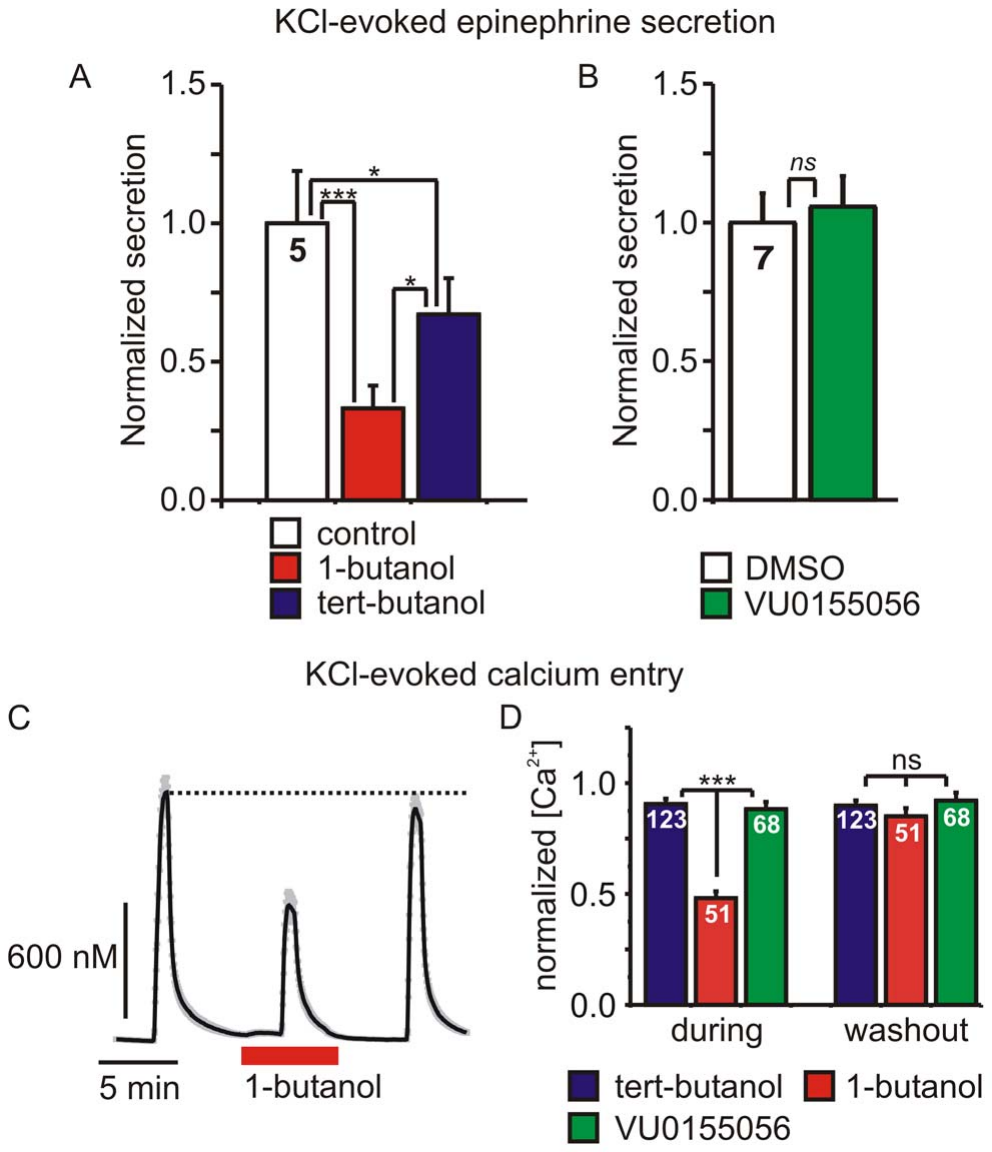
1-butanol but not the phospholipase D inhibitor VU0155056 inhibits catecholamine secretion and calcium entry. **A**) Secretion was evoked by application of 30 mM KCl for five-minutes. Epinephrine secretion from cells treated with 1-butanol (0.4%; ∼44 mM) or tert-butanol (0.4%; ∼42 mM) was normalized to that from controls. Secretion was significantly inhibited by 1-butanol and to a lesser extent by tert-butanol (one-way ANOVA, F = 20.98, p = 0.0007: * p<0.05, *** p<0.001 for pairwise comparisons using Tukey's post-hoc test). **B**) KCl-evoked epinephrine secretion was not altered by the phospholipase D inhibitor VU0155056 (1 µM) (p = 0.49, paired t-test). **C**) A representative experiment showing the intracellular calcium transients evoked by 30 mM KCl (60 s applications) applied before, during, and after washout of 0.4% 1-butanol. **D**) Mean data from several experiments like that shown in panel C. The peak increase in intracellular [Ca^2+^] evoked by KCl in the presence (during) of 1-butanol (0.4%; ∼42 mM), tert-butanol (0.4%; ∼42 mM), or VU0155056 (1 µM) was normalized to the first control response in each cell. 1-butanol significantly inhibited Ca^2+^ entry compared to both tert-butanol and VU0155056 (one-way ANOVA, F = 54.8, p<0.0001: *** p<0.001 for pairwise comparisons using Tukey's post-hoc test). After washout of the drugs (washout) Ca^2+^ entry was not significantly different in any of the three treatment groups (one-way ANOVA, F = 1.03, p = 0.36).

Dishes of chromaffin cells were stimulated with 30 mM KCl for 5 minutes to evoke membrane depolarization, calcium entry through voltage-gated calcium channels, and thus catecholamine secretion. Pre-incubation of the cells with 1-butanol (0.4%; ∼44 mM) dramatically reduced epinephrine secretion measured using HPLC (Fig 1A). Tert-butanol (0.4%; ∼42 mM) also reduced catecholamine secretion but to a significantly lesser extent than 1-butanol (Fig 1A). In contrast, 1 µM VU0155056, a newly developed cell membrane permeable inhibitor of both PLD1 and PLD2 [13], had no effect on secretion compared to DMSO controls (Fig 1B). These results suggest that 1-butanol exerts the bulk of its inhibitory effect on exocytosis at a site(s) other than PLD.

Because calcium entry through voltage-gated calcium channels plays a pivotal role in catecholamine secretion [36]–[39], we used ratiometric imaging of FURA2-loaded cells to determine if 1-butanol altered KCl-evoked calcium entry. Cells were stimulated with three successive applications of 30 mM KCl (Fig 1C). 1-butanol (0.4%; ∼44 mM) reversibly reduced the peak calcium transient by 52±3% (n = 51 cells: p<0.001, Tukey's post-test following repeated measures ANOVA). In contrast, only slight reductions in calcium entry were produced by tert-butanol (9±2%) or VU-155056 (12±3%), and these did not reverse upon washout of the drugs. In other experiments, we used longer preincubations with butanol or VU0155056 (10 - 15 minute preincubation) and measured the peak intracellular calcium transient evoked by a single application of KCl. The peak calcium transient in control cells was 1319±65 nM (n = 43) and was significantly smaller in cells treated with 0.4% 1-butanol (797±38 nM; n = 62; p<0.001 ANOVA with Tukey's post-test). In contrast, neither tert-butanol (1153±29 nM, n = 99) nor VU0155056 (1112±84 nM; n = 71) significantly reduced the peak calcium transient compared to controls (ANOVA followed by pair-wise comparison with Tukey's post-test).

### Differential effects of butanol isomers on voltage-gated calcium channel currents (*I_Ca_*)

**Figure 2:**
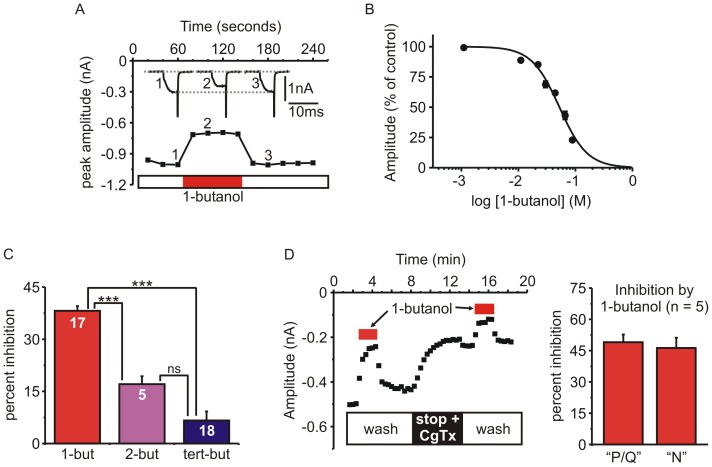
Concentration-dependent inhibition of *I_Ca_* by 1-butanol. **A**) Representative time-course showing reversible inhibition of peak *I_Ca_* amplitude by 1-butanol (0.4%; ∼44 mM). Currents were evoked by 10 ms duration steps from −90 mV to +10 mV. **B**) Concentration-response curve showing the percent inhibition of *I_Ca_* amplitude by 1-butanol. Points are mean ± s.e.m. of 4–17 cells (error bars are within the data points in some cases). The solid line shows the fit to a standard dose-response relationship which yielded an estimated EC_50_ of 52 mM and Hill slope of −1.73. **C**) Percent inhibition of peak *I_Ca_* amplitude (elicited by a step from −90 mV to +10 mV) by 1-butanol (n = 17), 2-butanol (n = 5), or tert-butanol (n = 18) (all butanol isomers at 0.4%) (one-way ANOVA, F = 60.7, p<0.0001: *** p<0.001, ns  =  “not significant” for pairwise comparisons using Tukey's post-hoc test). **D**) *Left panel*: Representative experiment showing peak *I_Ca_* amplitude plotted against time (elicited by a step from −70 mV to +10 mV). Nitrendipine (3 µM) was present throughout the experiment to block any L-type calcium channels. The bath was continually perfused with fresh solution (wash) and 1-butanol applied as indicated by the red bars. At the time indicated by the black bar, the solution flow was stopped and a bolus of ω-conotoxin GVIA was added to the bath (stop + CgTx) to irreversibly block N-type calcium channels. *Right panel*: The percent inhibition of pharmacologically isolated P/Q-type and N-type *I_Ca_* by 1-butanol. “P/Q-type” *I_Ca_* was defined as the CgTx resistant current (i.e. the current remaining after washout of CgTx). “N-type” *I_Ca_* was defined as the CgTx sensitive component (i.e. the data after Cgtx subtracted from data before CgTx).

Based on the calcium imaging data (Fig 1), we compared the effects of butanol isomers on *I_Ca_* using patch clamp electrophysiology. Cells were voltage-clamped in the standard whole-cell recording configuration and *I_Ca_* activated by short (10–20 ms) steps from −80 mV to +10 mV every 20 s (Fig 2A). Acute application of 1-butanol (0.4%; ∼44 mM) rapidly and reversibly inhibited the peak amplitude of *I_Ca_*. The inhibition of *I_Ca_* was concentration-dependent with an estimated EC_50_ of 52 mM (∼0.48%) (Fig 2B). We then compared the ability of 1-butanol, 2-butanol, and tert-butanol (all at 0.4%) to inhibit the peak amplitude of *I_Ca_*. 1-butanol inhibited *I_Ca_* from 640±88 pA to 399±57 pA (n = 17, p<0.0001 paired t-test); 2-butanol inhibited *I_Ca_* from 652±81 pA to 547±81 pA (n = 5; p<0.001 paired t-test); tert-butanol only slightly reduced the amplitude of *I_Ca_* from 632±83 pA to 583±78 pA (n = 18, p = 0.06 paired t-test). The percent inhibition by 1-butanol was significantly greater than either 2-butanol or tert-butanol (Fig 2C).

### 1-butanol inhibits N-type and P/Q-type *I_Ca_* to the same extent

The whole cell *I_Ca_* in bovine chromaffin cells has been well characterized and is comprised of ∼10% L-type current (blocked by dihydropyridine antagonists) with the remaining current carried by N-type and P/Q-type *I_Ca_* in roughly equal proportions [37], [40], [41]. To determine if the N- and P/Q-type channels were differentially inhibited by 1-butanol, we pharmacologically isolated the two current components. Cells were voltage-clamped at −70 mV and nitrendipine (3 µM) was present throughout the experiment to block the L-type current. Cells were stimulated every 20 s with a 20 ms step to +10 mV. The inhibition of *I_Ca_* produced by 1-butanol was determined twice, once before and once after application of ω-conotoxin GVIA (CgTx) to block the N-type channels (Fig 2D). Consistent with previous reports, CgTx blocked 48±7% (n = 5) of the current [37], [42], [43]. We have previously shown that the remaining CgTx resistant component of *I_Ca_* is almost fully comprised of P/Q-type channels, as it is blocked by ω-Agatoxin IVA [42]. Calculating the “difference current” (i.e. subtracting the data recorded after CgTx from that recorded before CgTx) yields the CgTx-sensitive N-type *I_Ca_*. Using this pharmacological dissection, we found that 1-butanol inhibited N-type *I_Ca_* by 46±5% and P/Q-type *I_Ca_* by 49±3.7% (n = 5) (Fig 2D).

### Differential effects of primary and tert-butanol on channel activation

**Figure 3:**
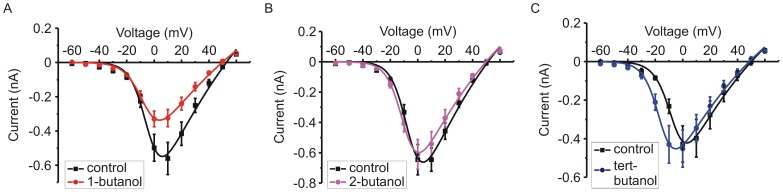
Differential effects of butanol isomers on the current-voltage relationship of *I_Ca_*. Cells were stimulated by a series of steps, from a holding potential of −90 mV to test potentials ranging from −60 mV to +60 mV, first in control conditions and then in the presence of either 0.4% (∼44 mM) 1-butanol (panel A, n = 6), 2-butanol (panel B, n = 5), or tert-butanol (panel C, n = 5). The mean current amplitude elicited by each step is plotted. Tert-butanol elicited a clear hyperpolarizing shift in the I-V relationship.

To assess the inhibition of *I_Ca_* in more detail, current-voltage relationships were determined. 1-butanol inhibited the amplitude of *I_Ca_* across a range of voltages with no obvious shift in the I-V curve (Fig 3A). In contrast, 2-butanol had much smaller effects (Fig 3B) and especially for tert-butanol there was an obvious hyperpolarizing shift in the I-V curve (Fig 3C). Indeed, the amplitude of *I_Ca_* elicited by a step to −10 mV was significantly *increased* by tert-butanol (p<0.01, repeated measures ANOVA with pairwise comparison using Bonferroni post-test).

**Figure 4:**
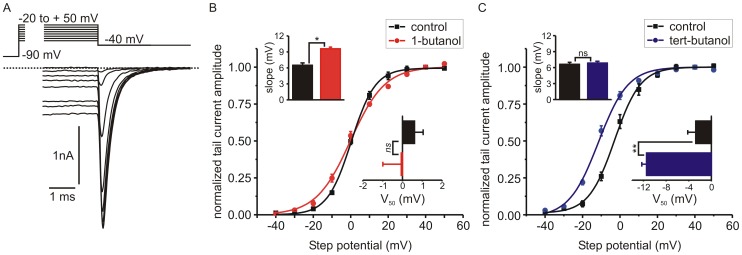
Butanol isomers differentially modulate the voltage-dependence of *I_Ca_* activation. **A**) The upper panel shows the voltage command protocol and the lower panel shows a family of representative *I_Ca_* tail current recordings. Cells were stimulated with depolarizing steps from a holding potential of −90 mV to test potentials ranging from −20 to +50 mV and tail currents were recorded upon repolarization to −40 mV. **B, C**) In each cell, tail currents were recorded first under control conditions and then in the presence of either 1-butanol (panel B, n = 6) or tert-butanol (panel C, n = 6). Tail current amplitude was normalized to that evoked by the +40 mV step and plotted against the potential of the step depolarization (mean ± s.e.m.). The solid line shows the fit with a Boltzmann curve. The inset bar graphs show the mean slope and V_50_ (potential at half maximal activation) derived from the Boltzmann fits to individual cells. 1-butanol had no effect on V_50_ but the slope of the curve was significantly shallower. In contrast, tert-butanol had no effect on the slope, but produced a strong hyperpolarizing shift in the V_50_ (* p<0.05; ** P<0.01; ns  =  not significant; paired t-test).

Given that tert-butanol produced a prominent shift in the I-V curve, we assessed the voltage-dependence of *I_Ca_* activation by measuring the tail current amplitude following a series of depolarizing steps (Fig 4A). Figures 4B and 4C show the effects of 1-butanol and tert-butanol on the activation curves. The V_50_ (potential at which half maximal activation is achieved) was determined by fitting a Boltzmann curve to the data. 1-butanol did not significantly shift the V_50_ for activation (0.6±0.4 mV in control conditions compared to −0.1±0.9 mV in the presence of 1-butanol, n = 6; p = 0.35, paired t-test). However, tert-butanol hyperpolarized the activation curve (Fig 4C) and significantly shifted the V_50_ from −2.7±1.4 mV to −11.4±0.8 (n = 6; p = 0.0018, paired t-test). The slope of the activation curve was significantly reduced by 1-butanol (Fig 4B) but not by tert-butanol (Fig 4C). Thus, the different butanol isomers exert distinct effects on channel activation which is opposed by 1-butanol but favored by tert-butanol.

### Opposite effects of primary and tert-butanol on the deactivation kinetics of *I_Ca_*

**Figure 5:**
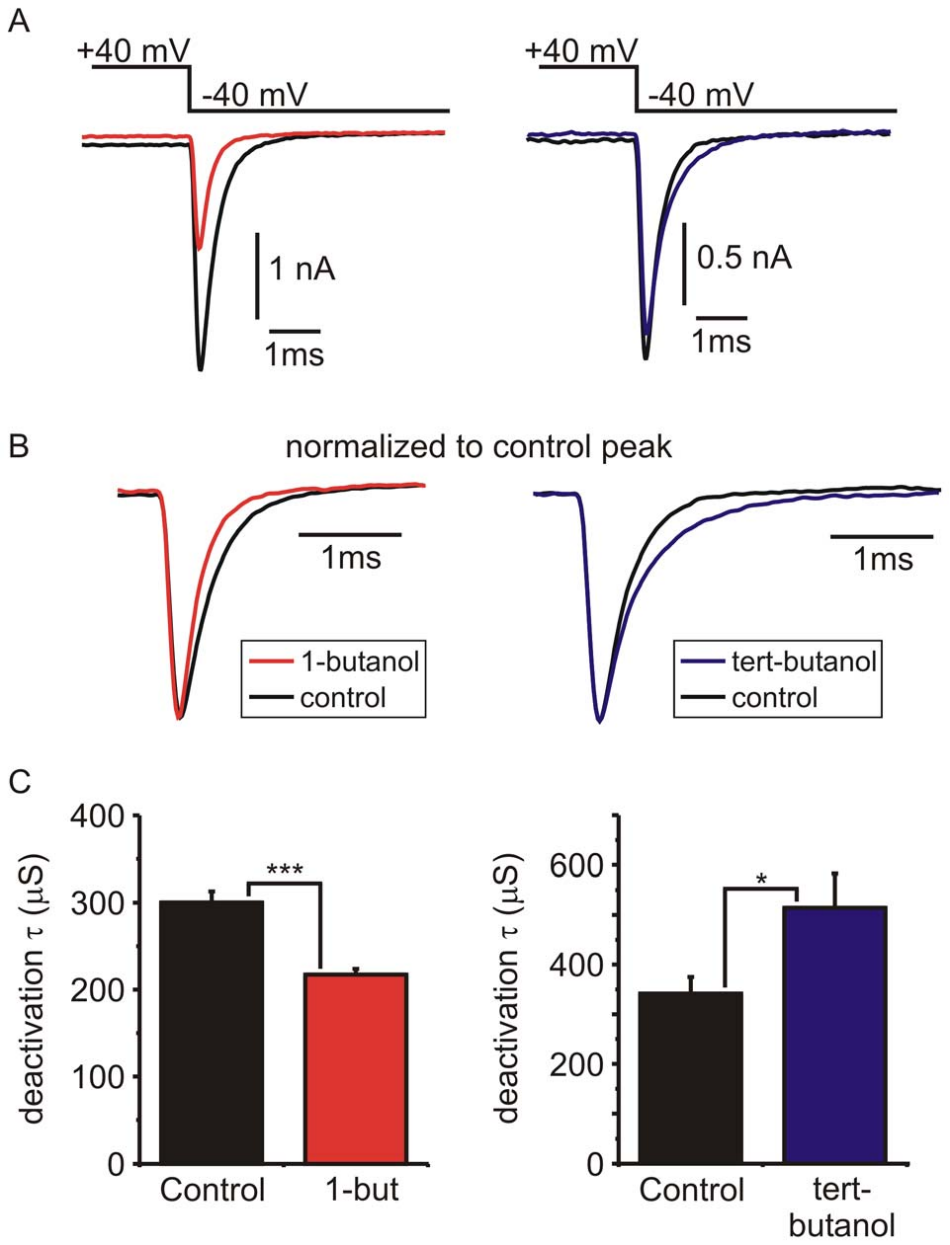
Differential effects of butanol isomers on the deactivation kinetics of *I_Ca_*. **A**) Representative tail currents activated by a step depolarization from −90 mV to +40 mV. In each cell, tail currents were recorded first in control conditions and then in the presence of either 1-butanol (left) or tert-butanol (right). **B**) The same traces as in panel A, but the time-base was expanded and the currents in the presence of butanol were scaled to the same amplitude as control to more clearly illustrate changes in the decay kinetics. **C**) The decay of the tail currents was fit with a single exponential function. Bar charts plot the mean time constant of the fit (τ) in control conditions and in the presence of 1-butanol (n = 6) or tert-butanol (n = 6). (* p<0.05; *** p<0.001 compared to matched controls; paired t-test).

Figure 5A shows examples of tail currents following steps to +40 mV to maximally activate *I_Ca_*. The mean tail current amplitude was significantly reduced by 1-butanol (from 1.81±0.33 nA to 0.92±0.15 nA; n = 6; p = 0.006, paired t-test) but not by tert-butanol (1.17±0.22 nA to 1.01±0.15 nA; n = 6; p = 0.14, paired t-test). The decay of the tail currents reflects channel deactivation (i.e. the open to closed transition upon membrane hyperpolarization) and is well fit with a single exponential. 1-butanol significantly accelerated the decay (Fig 5B, C), decreasing the time constant of the exponential fit from 300±13 µS to 217±7 µS (n = 6, p = 0.004, paired t-test) (Fig 5C). In contrast, tert-butanol significantly slowed the decay of the tail currents, increasing the time constant of the exponential fit from 340±33 µS to 514±68 µS (n = 6, p = 0.03, paired t-test) (Fig 5B, C).

### Primary and tert-butanol both promote voltage-dependent inactivation of *I_Ca_*

**Figure 6:**
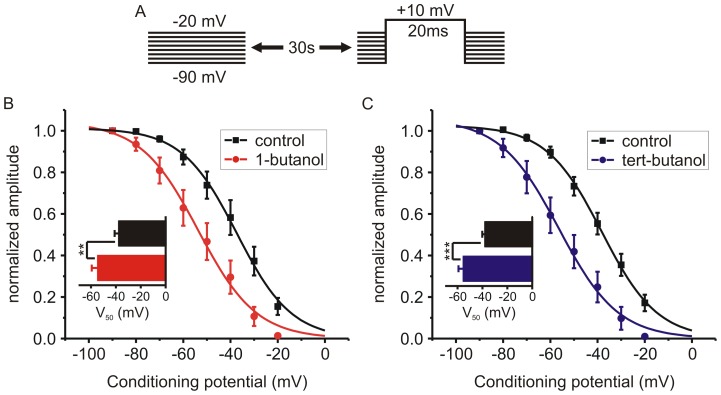
1-butanol and tert-butanol produce a similar hyperpolarization of closed-state inactivation. **A**) Schematic depiction of the voltage-stimulus protocol. *I_Ca_* were elicited by steps to +10 mV. The holding potential was increased in 10 mV increments from −90 mV to −20 mV and maintained at each potential for 30 s. In each cell this was done first in control conditions and then in the presence of either 1-butanol (panel B; n = 5) or tert-butanol (panel C; n = 6). **B, C**) *I_Ca_* amplitude was normalized to that evoked from a holding potential of −90 mV and plotted against the holding potential. The graphs show the mean data fit using a Boltzmann function with three free parameters (upper plateau, slope, and V_50_) and the lower plateau was constrained to ≥0. The inset bar graphs show the hyperpolarizing shift in mean V_50_ (voltage at half maximal inactivation) derived from the Boltzmann fits to individual cells (** p<0.01; *** p<0.001 compared to matched controls; paired t-test).

The voltage-dependence of steady-state inactivation was determined by incrementally increasing the holding potential from −90 mV to −20 mV for durations of 30 s (Fig 6A). Following recovery at −90 mV the cell was then exposed to either 1-butanol (0.4%; ∼44 mM) or tert-butanol (0.4%; ∼42 mM) and the inactivation protocol was repeated. Both butanol isomers produced a similar hyperpolarizing shift in the inactivation curves (Fig 6B, C). 1-butanol significantly shifted the V_50_ (potential at half maximal inactivation) from −37.4±3.6 mV to -54.3±5.1 mV (n = 5, p = 0.0016, paired t-test). Similarly, tert-butanol shifted the V_50_ from -37.8±2.4 mV to −55.1±4.2 mV (n = 6, p = 0.0006, paired t-test).

**Figure 7:**
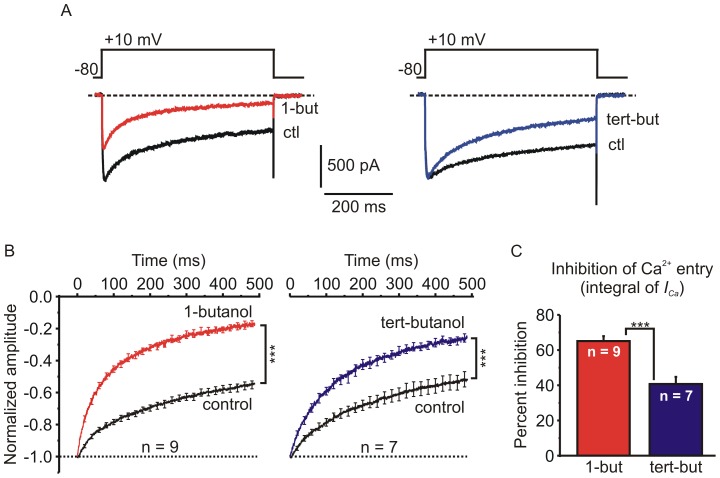
Butanol accelerates voltage-dependent inactivation and reduces total calcium entry during sustained depolarization. **A**) Representative recordings of *I_Ca_* elicited by a 500 ms step depolarization under control conditions (ctl) and in the presence of either 0.4% (∼44 mM) 1-butanol (1-but) or 0.4% (∼42 mM) tert-butanol (tert-but). The patch pipette solution contained 10 mM EGTA to block calcium-dependent inactivation. Thus, the decay in *I_Ca_* is due to voltage-dependent inactivation. 1-butanol inhibited the peak amplitude and accelerated the the inactivation of *I_Ca_*. Tert-butanol also accelerated inactivation, but had minimal effects of the peak amplitude of *I_Ca_*. **B**) Pooled data (mean ± sem) from multiple experiments like that shown in panel A. To facilitate comparison of inactivation, the amplitude of *I_Ca_* in the presence of butanol was normalized to control conditions. For clarity, the displayed traces begin at the peak amplitude and error bars are only shown for some data points. 1-butanol and tert-butanol both significantly increased the extent of inactivation. (*** p<0.001 for the extent of inactivation at 490 ms in the presence of butanol compared to control conditions; paired t-test). **C**) The charge (integral) of *I_Ca_* is directly proportional to the amount of calcium entry during the depolarizing step. 1-butanol produced a significantly greater inhibition than tert-butanol (*** p<0.001; paired t-test).

The effect of butanol on voltage-dependent inactivation during a sustained depolarizing step was also determined (Fig 7A, B). EGTA (10 mM) was present in the patch-pipette solution to block Ca^2+^-dependent inactivation. Cells were stimulated by a voltage step to +10 mV lasting 500 ms before and during application of either 1-butanol (0.4%; ∼44 mM) or tert-butanol (0.4%; ∼42 mM). Both butanol isomers significantly increased inactivation during the 500 ms step (Fig 7B). Accelerated inactivation will reduce calcium entry into cells during sustained or repetitive stimulation. To quantify this we calculated the charge (i.e. the integral) of *I_Ca_* evoked by a 500 ms step depolarization. 1-butanol significantly inhibited the charge of *I_Ca_* from 328±35 pC to 116±14 pC (n = 9; p<0.0001, paired t-test). Even though tert-butanol has little effect on the peak amplitude, it significantly reduced the charge of *I_Ca_* from 297±48 pC to 184±40 pC (n = 7; p<0.001, paired t-test). Reflecting the dual actions of 1-butanol on both peak amplitude and inactivation, the integral of *I_Ca_* was inhibited to a greater extent by 1-butanol (65±3%, n = 9) than by tert-butanol (41±4%, n = 7; p<0.001, independent t-test).

## Discussion

In this study we investigated the effects of butanol isomers on calcium signaling and catecholamine secretion from adrenal chromaffin cells, an important component of the sympathetic nervous system and powerful model for investigating neurosecretory mechanisms. The rationale for the current investigation arose from two general uses of butanol in cellular physiology: implication of phosphatidic acid/PLD signaling in cellular functions, and investigating the molecular targets of general anesthetics. In spite of these two uses, there is little data comparing the effects of butanol isomers on pertinent cellular targets including voltage-gated ion channels.

Our data show that 1-butanol inhibited secretion to a much greater extent than tert-butanol, but the PLD inhibitor VU0155056 had no effect (Fig 1). We used VU0155056 at 1 µM, a concentration well above the reported cellular IC_50_ for PLD1 (20 nM) and PLD2 (380 nM) [13], [14]. Our data parallel a previous report showing that 1-butanol inhibits insulin secretion but the PLD inhibitor FIPI has no effect [12]. Similarly, 1-butanol but not FIPI blocks degranulation (exocytosis) of mast cells [15]. Together, our data and these previous studies imply that acute application of 1-butanol blocks exocytosis by a PLD-independent mechanism(s). Our data demonstrate that disrupted calcium signaling is a likely mechanism by which 1-butanol acts. Using cellular imaging, we show that the calcium transient evoked by KCl-mediated depolarization was significantly reduced by 1-butanol, while tert-butanol and VU0155056 had negligible effects (Fig 1). Furthermore, we used patch clamp electrophysiology to confirm that 1-butanol rapidly and reversibly inhibited the peak amplitude of *I_Ca_* while secondary and tert-butanol had minimal effects (Fig 2).

Ten genes encode for the pore-forming α_1_ subunit of voltage-gated calcium channels, and these are sub-divided into three major families based on sequence homology (Ca_V_1, Ca_V_2, Ca_V_3) [44], [45]. Chromaffin cells from adult animals typically express Ca_V_1 and Ca_V_2 channels with the relative expression level depending on the species [37], [39]–[41]. Low threshold T-type channels (Ca_V_3) are typically not functionally expressed under normal conditions, but can be recruited by various signaling pathways or hypoxia [46]–[49]. In bovine cells, we have previously demonstrated that the whole cell *I_Ca_* is comprised of ∼10% L-type current and ∼90% N + P/Q-type current in a roughly 1∶1 ratio [42], [50]. The concentration response relationship to 1-butanol (Fig 2B) shows that higher concentrations almost fully blocked *I_Ca_*, suggesting that all the calcium channel subtypes present in these cells were targeted. To confirm this, we pharmacological dissected *I_Ca_* using ω-conotoxin GVIA (a selective blocker of N-type channels) and found that the N-type and P/Q-type *I_Ca_* were equally blocked by 1-butanol (Fig 2D). We speculate that L-type channels will be modulated in a similar manner, but due to the small amplitude of the L-type current in bovine chromaffin cells we did not determine this. In rodent chromaffin cells L-type channels (Ca_V_1.2 and Ca_V_1.3) comprise ∼40% of the whole cell calcium current, and have been implicated in controlling action potential firing at rest or during sustained depolarization [51]. It is interesting to speculate that the butanol isomers will differentially modulate these aspects of rodent chromaffin cell function, but further experiments are needed to confirm this.

Our data showing isomer-specific block of *I_Ca_* by butanol suggests the need to reassess at least some cellular functions previously attributed to PLD signaling using primary alcohols, especially those in which calcium signaling plays a prominent role. There is a body of work supporting a role for PLD in calcium-dependent exocytosis from chromaffin or PC12 cells [9], [11], [34], although some other studies have found no such link [52]–[54]. While the precise mechanism remains unclear, it has been proposed that generation of phosphatidic acid at vesicle release sites induces membrane curvature and thereby promotes the final stages of vesicle fusion with the plasma membrane [11]. Phosphatidic acid has also been shown to interact directly with SNARE proteins [55], or could lead to changes in other signaling lipids such as diacylglycerol or PiP2 [10]. Several approaches including RNAi-mediated knockdown of PLD and overexpression of wild-type or dominant negative mutants have been used to implicate PLD, and in particular PLD1, in exocytosis (for reviews see [9], [11]). However, acute interventions have typically involved the use of primary alcohols, and to our knowledge the effects of newer PLD inhibitors have not been reported prior to this study. The main focus of the current investigation was not to dissect the role of PLD in catecholamine secretion *per se*, but our data suggest that acute block of PLD does not have a dramatic impact. Subtle effects on secretion are still possible as these would not be resolved in the assays used in this study, and we did not assess the impact of longer term block of PLD signaling. Further investigations will be required to address these issues and the precise role of PLD in neurotransmitter/hormone secretion.

This paper also provides the first detailed comparison of how butanol isomers modulate the function of voltage-gated channels. We used a concentration of 0.4% (∼44 mM), typical for experiments probing PLD signaling and close to the EC_50_ for block of peak *I_Ca_* amplitude by 1-butanol (Fig 2B). Although tert-butanol had minimal effects on peak *I_Ca_* amplitude, it was not simply inert with respect to channel function. Tert-butanol accelerated inactivation of *I_Ca_* during sustained depolarization and produced a pronounced (approximately −20 mV) shift in the steady state inactivation curve (Figs 6, 7). Distinct, even opposing effects of the butanol isomers were seen on other channel gating parameters, including the voltage-dependence of activation. 1-butanol produced no shift in the V_50_ and a modest reduction in the slope of the activation curve (Fig 4B). In contrast, tert-butanol hyperpolarized the V_50_ by ∼−9 mV, with no change in the slope (Fig 4C). This likely will not increase the small “window current” (overlap of activation and inactivation curves (Figs 4 and 6)) because the much larger shift in steady-state inactivation will dominate. However, the hyperpolarizing shift in activation was apparent in the I-V relationship, and resulted in tert-butanol significantly *potentiating* the amplitude of *I_Ca_* evoked by steps to −10 mV (Fig 3). The two isomers also had opposing effects on the kinetics of deactivation (tail current decay), which was accelerated by 1-butanol and slowed by tert-butanol (Fig 5). Again, as with the effects on activation gating, this would favor a potentiation of *I_Ca_* by tert-butanol and inhibition by 1-butanol.

Perhaps the complex effects of butanol on *I_Ca_* reflect multiple binding sites on the channel, some of which discern between the structurally distinct primary and tert-butanol isomers. 1-butanol has been used to map a binding site for general anesthetics/alcohols near the activation gate of Shaw-2 voltage-gated potassium channels [28], [29]. The effects we observe on activation and deactivation gating are consistent with there being a similar binding site for butanol on the voltage-gated calcium channels, although this remains to be determined. Another recent study has reported multiple binding sites for general anesthetics on the bacterial sodium channel NaChBac [56]. It is also interesting to note that in virtually all cases reported to date, alcohols/general anesthetics *inhibit* voltage-gated channels. However, sevoflurane can potentiate voltage-gated potassium channels [57] and NaChBac [56]. It will be of interest in future to determine if sevoflurane also potentiates Ca_V_ channels, and conversely if tert-butanol potentiates potassium and sodium channels.

Although much progress has been made, the molecular targets and mechanisms of general anesthetics/alcohols are not completely understood. There is general consensus that post-synaptic ligand gated ion channels (e.g GABA, ACh) are important targets, but some evidence also points to presynaptic effects, perhaps on voltage-gated channels or the exocytotic machinery itself including SNARE proteins and munc18 [58]–[61]. In this study the estimated EC_50_ for 1-butanol block of peak *I_Ca_* amplitude was ∼52 mM (Fig 2B), which is somewhat higher than that producing anesthesia in vivo (10–20 mM) [62]–[64]. Moreover, even though secondary and tert-butanol also produce anesthesia, they had minimal effects on *I_Ca_* amplitude (Fig 2,3). This would argue against Ca_V_2 voltage-gated calcium channels being a primary target for the anesthetic actions. However, even relatively small changes in *I_Ca_* can have a significant impact on neurotransmitter release, and as already discussed the effects of butanol are complex and extend well beyond the simple block of peak current amplitude. Further, investigations will be required to determine if these channels contribute to some of the anesthetic actions and/or side effects of alcohols/inhalational anesthetics.

In conclusion, we show for the first time that butanol isomers have distinct effects on the amplitude and gating properties of Ca_V_2 voltage-gated calcium channel currents. This adds to existing literature demonstrating complex modulation of voltage-gated channels by alcohols and anesthetic agents. Our data also suggest the need to reassess at least some cellular functions previously attributed to PLD signaling by using primary alcohols as a tool, especially those in which calcium signaling plays a prominent role.

## Supporting Information

Data S1 This file is provided as part of the PLOS ONE data availability policy. For figures in the manuscript that present averaged data (e.g. mean ± sem), the underlying raw data used to calculate those averages are provided. Refer to the main text of the manuscript or figure legends for further explanation of experimental design, protocols, etc.
(PDF)
