# Supplementary material for: Butanol Isomers Exert Distinct Effects on Voltage-Gated Calcium Channel Currents and Thus Catecholamine Secretion in Adrenal Chromaffin Cells
Source: PLoS One. 2014 Oct 2;9(10):e109203. doi: 10.1371/journal.pone.0109203 (PMC4183593; doi:10.1371/journal.pone.0109203)
Supplement: Data S1 — This file is provided as part of the PLOS ONE data availability policy. For figures in the manuscript that present averaged data (e.g. mean ± sem), the underlying raw data used to calculate those averages are provided. Refer to the main text of the manuscript or figure legends for further explanation of experimental design, protocols, etc. (PDF) [file pone.0109203.s001.pdf]

DOI: 10.1371/journal.pone.0109203

Butanol isomers exert distinct effects on voltage-gated calcium channel currents and thus catecholamine secretion in adrenal chromaffin cells

Sarah McDavid, Mary Beth Bauer, Rebecca L. Brindley, Mark L. Jewell, and Kevin P.M. Currie

The following data is provided as part of the PLOS ONE data availability policy. For figures in the manuscript that present averaged data, the underlying raw data used to calculate those averages are provided.

For further explanation of the experimental protocols etc refer to the main text of the manuscript.

Figure 1A (normalized epinephrine secretion)		
Control	1-butanol	tert-butanol
0.91	0.22	0.42
1.21	0.53	1.07
0.70	0.25	0.56
1.62	0.52	0.89
0.56	0.13	0.42

Figure 1B (normalized secretion)	
DMSO	VU0155056
1.10	1.17
1.15	1.12
1.25	1.31
0.88	1.37
1.33	1.13
0.62	0.67
0.67	0.64

Figure 1D (normalized calcium increase)					
during drug application			washout of drug		
**tert-butanol**	**1-butanol**	**VU0155056**	**tert-butanol**	**1-butanol**	**VU0155056**
0.487	0.158	0.391	0.382	0.533	0.228
0.499	0.254	0.638	0.513	0.253	0.414
0.844	0.417	0.340	0.812	0.606	0.195
0.571	0.633	0.637	0.506	0.698	0.559
0.514	0.197	0.721	0.457	0.328	0.486
0.477	0.294	0.778	0.467	0.379	0.595
0.591	0.833	0.462	0.153	0.947	0.305
0.684	0.181	0.530	0.558	0.372	0.862
0.574	0.261	1.123	0.523	0.655	1.097
0.543	0.377	0.528	0.488	0.699	0.620
0.449	0.271	0.646	0.454	0.708	0.656
0.483	0.157	0.862	0.578	0.422	1.136
0.466	0.478	1.231	0.580	0.674	1.247
0.693	0.166	1.058	0.564	0.735	1.112
0.696	0.394	0.904	0.707	0.713	0.906
0.719	0.474	0.299	0.534	0.736	0.111
0.068	0.544	0.583	0.537	0.647	0.940
0.739	0.825	0.866	0.636	1.060	0.947
0.601	0.357	1.005	0.559	0.786	1.008
0.820	0.458	0.824	0.830	0.804	1.070
1.121	0.393	0.882	1.100	0.684	0.823

0.633	0.359	0.875	0.699	0.552	0.936
0.874	0.282	0.763	0.874	0.898	0.710
0.718	0.474	0.466	0.607	0.705	0.547
0.773	0.445	0.876	0.751	0.924	0.960
0.672	0.295	0.722	0.713	0.808	1.009
0.681	0.484	0.950	0.582	0.839	1.073
0.648	0.339	0.801	0.698	0.884	0.970
0.657	0.525	0.802	0.694	1.021	0.885
0.760	0.657	1.297	0.765	0.902	1.342
0.715	0.783	0.773	0.686	0.945	0.879
0.753	0.644	0.883	0.663	0.920	1.090
0.846	0.424	1.171	0.847	0.996	1.212
1.161	0.345	1.306	1.050	1.151	1.175
0.792	0.366	1.158	0.781	0.679	1.285
0.959	0.561	0.772	1.038	0.958	0.874
0.788	0.360	0.775	0.731	0.976	0.908
0.798	0.760	0.969	0.726	1.063	0.920
0.677	0.556	0.539	0.750	0.961	0.583
0.646	0.366	1.024	0.746	1.068	1.151
0.761	0.834	0.668	0.733	1.025	0.901
0.817	0.788	1.051	0.772	1.245	1.161
0.914	0.732	0.899	0.905	1.009	0.932
0.808	0.239	0.955	0.767	1.031	1.054
0.896	0.562	0.884	0.787	0.945	0.932
0.990	0.735	1.442	0.954	1.013	1.465
1.096	0.510	1.175	0.840	1.183	1.259
0.753	0.650	0.843	0.786	1.070	0.958
0.793	1.024	0.950	0.776	1.201	0.986
0.909	0.407	1.320	0.898	1.515	1.305
0.810	0.923	0.747	0.788	1.419	0.555
0.841		1.309	0.806		1.360
0.839		0.686	0.810		0.812
0.763		1.173	0.794		1.210
1.082		0.741	1.081		1.046
0.643		0.792	0.811		0.926

0.659	1.299	0.858	1.400
0.903	1.099	0.835	1.080
0.717	0.894	0.827	0.749
0.731	0.951	0.867	0.920
0.867	1.420	0.774	1.450
1.178	1.118	1.036	1.132
0.861	0.816	0.704	0.826
0.834	0.403	0.713	0.394
0.895	0.926	0.878	0.921
0.777	0.989	0.859	0.970
0.739	1.003	0.764	0.909
1.139	1.254	1.073	1.178
0.610		0.834	
1.028		1.045	
0.657		0.819	
0.929		0.852	
0.982		0.984	
0.990		0.822	
0.932		0.953	
1.178		1.142	
0.882		0.888	
1.016		1.098	
1.017		0.992	
1.012		0.833	
1.030		0.740	
1.102		1.107	
1.127		0.933	
1.181		1.240	
0.734		0.923	
0.842		1.054	
0.961		0.914	
0.979		0.932	
1.057		1.017	
0.918		0.971	
0.982		0.970	

0.760	0.959
1.223	1.204
1.125	1.219
0.859	1.033
1.384	1.321
1.137	1.158
1.105	1.037
1.274	1.310
1.205	1.099
0.883	1.074
1.194	1.140
1.566	1.293
1.262	1.095
1.232	1.232
1.172	1.144
1.220	1.301
1.244	1.243
0.929	1.199
1.185	1.099
0.935	1.023
1.225	1.147
1.497	1.472
1.087	1.314
1.007	1.199
1.402	1.355
1.302	1.326
1.312	1.314
1.142	1.209
1.260	1.153
1.169	1.308
1.449	1.330
1.358	1.367

Fig 2B concentration response (normalized current amplitude)

concentration of 1-butanol	0.01%	0.10%	0.20%	0.28%	0.40%	0.60%	0.80%
	1.020	0.888	0.887	0.683	0.522	0.469	0.254
	0.990	0.916	0.812	0.769	0.716	0.334	0.243
	1.010	0.905	0.840	0.677	0.636	0.473	0.150
	0.980	0.886	0.885	0.608	0.668	0.453	0.249
	0.950	0.892	0.831	0.707	0.608		0.253
	1.010	0.865	0.876		0.598		
		0.864	0.832		0.604		
		0.887			0.617		
		0.873			0.603		
		0.903			0.655		
					0.541		
					0.543		
					0.619		
					0.595		
					0.591		
					0.680		
					0.711		

Fig 2C (% inhbition of Ica by butanol isomers)

1-butanol	2-butanol	tert butanol
45.7	20.1	-5.7
38.1	24.2	0.6
40.5	15.7	3.4
40.9	11.6	-6.7
32.0	13.9	19.8
28.9		34.0
47.8		8.5
28.4		2.0
36.4		19.9
33.2		-11.5
39.2		1.8
40.2		15.6
39.6		4.0
38.3		15.4
39.7		4.8
34.5		2.4
45.9		10.3
0.6		

Fig 2D: % inhbition of P/Q type and N-type Ica by 1-butanol

P/Q-type	N-type
61.1	41.9
40.9	36.8
53.7	36.5
45.4	58.7
44.2	57.4

Figure 3A - current - voltage relationship with 1-butanol

	control amplitude (Amps)						1-butanol amplitude (Amps)					
X axis (mV)	cell-1	2	3	4	5	6	cell-1	2	3	4	5	6
-60	-1.58E-11	8.80E-12	-9.86E-12	-9.45E-12	-8.34E-12	-9.24E-12	-1.15E-11	1.50E-11	-2.68E-11	1.16E-11	8.85E-12	8.54E-12
-50	1.14E-11	-1.00E-11	-7.61E-12	8.20E-12	-9.90E-12	-2.21E-11	-1.03E-11	-1.42E-11	-9.16E-12	-1.13E-11	-6.47E-12	-1.63E-11
-40	-2.32E-11	-1.18E-11	-1.35E-11	-1.69E-11	-2.07E-11	-7.56E-11	-1.84E-11	-1.06E-11	-1.11E-11	-1.45E-11	-1.09E-11	-1.99E-11
-30	-5.82E-11	-3.02E-11	-4.31E-11	-7.40E-11	-4.51E-11	-4.98E-11	-4.45E-11	-2.28E-11	-6.20E-11	-3.25E-11	-2.18E-11	-3.27E-11
-20	-1.05E-10	-7.80E-11	-1.04E-10	-1.21E-10	-5.47E-11	-5.29E-11	-9.57E-11	-6.85E-11	-9.43E-11	-7.64E-11	-4.10E-11	-5.36E-11
-10	-2.47E-10	-2.61E-10	-3.14E-10	-2.98E-10	-1.04E-10	-1.32E-10	-2.05E-10	-2.24E-10	-2.60E-10	-2.36E-10	-9.50E-11	-1.32E-10
0	-4.43E-10	-6.86E-10	-5.92E-10	-7.00E-10	-2.37E-10	-3.29E-10	-2.79E-10	-4.56E-10	-3.83E-10	-4.42E-10	-2.01E-10	-2.16E-10
10	-4.51E-10	-8.42E-10	-6.21E-10	-7.99E-10	-3.04E-10	-3.44E-10	-2.57E-10	-4.68E-10	-3.55E-10	-4.63E-10	-2.19E-10	-1.84E-10
20	-3.33E-10	-6.06E-10	-4.46E-10	-6.11E-10	-2.34E-10	-2.64E-10	-1.81E-10	-3.37E-10	-2.54E-10	-3.60E-10	-1.61E-10	-1.50E-10
30	-1.99E-10	-3.55E-10	-2.65E-10	-3.82E-10	-1.35E-10	-1.68E-10	-1.02E-10	-1.95E-10	-1.54E-10	-2.23E-10	-9.39E-11	-7.99E-11
40	-1.05E-10	-1.61E-10	-1.24E-10	-1.91E-10	-5.29E-11	-8.62E-11	-4.43E-11	-6.63E-11	-7.44E-11	-1.09E-10	-3.72E-11	-3.49E-11
50	-3.39E-11	-1.98E-11	-3.36E-11	-5.72E-11	1.75E-11	-2.83E-11	1.06E-11	4.51E-11	-1.36E-11	-2.80E-11	2.01E-11	1.17E-11
60	3.07E-11	8.92E-11	3.89E-11	4.99E-11	5.03E-11	2.96E-11	3.90E-11	9.60E-11	3.87E-11	4.34E-11	4.87E-11	-8.42E-11

Figure 3B - current - voltage relationship with 2-butanol

	control amplitude (Amps)					2-butanol amplitude (Amps)				
X axis (mV)	cell-1	2	3	4	5	cell-1	2	3	4	5
-60	-1.28E-11	-1.47E-11	-8.27E-12	-1.35E-11	-1.36E-11	-1.29E-11	-1.27E-11	5.18E-12	-1.29E-11	-1.37E-11
-50	1.35E-11	-2.64E-11	-1.13E-11	-1.25E-11	1.39E-11	1.73E-11	-2.35E-11	-1.13E-11	-1.31E-11	-1.74E-11
-40	-1.91E-11	-4.96E-11	-1.44E-11	-1.70E-11	-2.35E-11	-2.28E-11	-4.06E-11	-1.84E-11	-2.64E-11	-2.06E-11
-30	-4.52E-11	-8.80E-11	-5.13E-11	-4.88E-11	-5.17E-11	-3.66E-11	-6.42E-11	-4.91E-11	-5.24E-11	-4.23E-11
-20	-9.89E-11	-1.06E-10	-1.18E-10	-1.89E-10	-8.55E-11	-9.17E-11	-1.74E-10	-1.76E-10	-2.00E-10	-1.13E-10
-10	-2.42E-10	-3.04E-10	-3.63E-10	-5.10E-10	-2.42E-10	-2.79E-10	-4.93E-10	-3.49E-10	-5.70E-10	-3.46E-10
0	-4.76E-10	-5.78E-10	-5.60E-10	-1.04E-09	-5.77E-10	-4.45E-10	-6.47E-10	-4.12E-10	-9.60E-10	-5.88E-10
10	-5.45E-10	-5.94E-10	-5.17E-10	-9.43E-10	-6.28E-10	-4.47E-10	-5.58E-10	-3.45E-10	-8.30E-10	-5.27E-10
20	-4.19E-10	-3.86E-10	-3.63E-10	-6.66E-10	-4.52E-10	-3.31E-10	-3.69E-10	-2.30E-10	-5.77E-10	-3.52E-10
30	-2.61E-10	-2.44E-10	-2.05E-10	-3.83E-10	-2.61E-10	-1.99E-10	-2.03E-10	-1.17E-10	-3.34E-10	-2.01E-10
40	-1.24E-10	-1.08E-10	-9.61E-11	-1.68E-10	-1.13E-10	-9.53E-11	-7.84E-11	-5.65E-11	-1.29E-10	-9.21E-11
50	-4.01E-11	1.92E-11	-2.11E-11	2.62E-11	-2.55E-11	-2.34E-11	3.07E-11	-1.42E-11	3.32E-11	-8.16E-12
60	4.09E-11	4.68E-11	4.70E-11	1.36E-10	6.90E-11	4.50E-11	1.01E-10	3.31E-11	1.34E-10	6.19E-11

Figure 3C - current - voltage relationship with tert-butanol										
	control amplitude (Amps)					tert-butanol amplitude (Amps)				
X axis (mV)	cell-1	2	3	4	5	cell-1	2	3	4	5
-60	-1.13E-11	-7.43E-12	1.12E-11	-1.27E-11	6.99E-12	-1.37E-11	-8.20E-12	-1.14E-11	-1.34E-11	-1.02E-11
-50	9.71E-12	-9.31E-12	-1.61E-11	-7.81E-12	-1.00E-11	-1.35E-11	-1.12E-11	-2.50E-11	-1.75E-11	-1.61E-11
-40	-1.03E-11	-1.51E-11	-3.20E-11	-1.37E-11	-3.04E-11	-2.38E-11	-1.90E-11	-4.91E-11	-1.99E-11	-5.04E-11
-30	-2.08E-11	-3.18E-11	-8.66E-11	-2.80E-11	-8.76E-11	-6.11E-11	-5.37E-11	-9.49E-11	-3.68E-11	-1.19E-10
-20	-7.23E-11	-9.34E-11	-8.68E-11	-5.82E-11	-1.14E-10	-2.82E-10	-1.21E-10	-2.44E-10	-1.01E-10	-3.06E-10
-10	-2.89E-10	-1.64E-10	-2.93E-10	-1.21E-10	-2.92E-10	-6.81E-10	-2.07E-10	-5.09E-10	-1.94E-10	-5.56E-10
0	-6.90E-10	-2.36E-10	-5.13E-10	-1.87E-10	-5.12E-10	-7.02E-10	-2.34E-10	-5.38E-10	-2.16E-10	-5.67E-10
10	-7.04E-10	-2.07E-10	-4.39E-10	-1.67E-10	-4.71E-10	-5.26E-10	-1.93E-10	-4.25E-10	-1.67E-10	-4.14E-10
20	-5.10E-10	-1.38E-10	-2.94E-10	-1.10E-10	-3.29E-10	-3.64E-10	-1.25E-10	-2.68E-10	-1.18E-10	-2.79E-10
30	-3.21E-10	-7.10E-11	-1.64E-10	-6.66E-11	-1.81E-10	-2.13E-10	-6.18E-11	-1.49E-10	-6.68E-11	-1.45E-10
40	-1.66E-10	-1.94E-11	-6.82E-11	-3.08E-11	-7.63E-11	-1.12E-10	-1.82E-11	-5.88E-11	-2.99E-11	-5.54E-11
50	-5.59E-11	3.28E-11	1.55E-11	-1.95E-11	2.34E-11	-3.24E-11	3.04E-11	2.91E-11	1.36E-11	2.48E-11
60	3.65E-11	5.86E-11	8.20E-11	3.35E-11	7.33E-11	3.54E-11	5.03E-11	7.62E-11	3.36E-11	6.84E-11

Figure 4B and C: Activation curves (normalized tail current amplitude Vs voltage-step potential), and data extracted from Boltzmann fits

Figure 4B (1-butanol data)

	control normalized tail amplitude						1-butanol normalized tail amplitude					
X axis (mV)	cell-1	2	3	4	5	6	cell-1	2	3	4	5	6
-40	0.011	0.002	0.004	0.004	0.014	0.043	0.011	0.003	0.006	0.004	0.009	0.025
-30	0.034	0.011	0.018	0.017	0.048	0.011	0.046	0.016	0.049	0.021	0.014	0.041
-20	0.018	0.028	0.049	0.044	0.060	0.041	0.121	0.013	0.101	0.063	0.046	0.118
-10	0.178	0.109	0.185	0.141	0.137	0.149	0.329	0.180	0.288	0.219	0.178	0.292
0	0.488	0.444	0.517	0.459	0.486	0.585	0.562	0.489	0.572	0.492	0.459	0.641
10	0.754	0.797	0.791	0.764	0.930	0.813	0.771	0.714	0.757	0.734	0.719	0.800
20	0.889	0.918	0.914	0.899	1.053	0.970	0.864	0.866	0.865	0.841	0.854	0.920
30	0.912	0.974	0.963	0.976	1.064	1.017	0.953	0.937	0.957	0.972	0.945	0.941
40	1.000	1.000	1.000	1.000	1.000	1.000	1.000	1.000	1.000	1.000	1.000	1.000
50	1.016	0.998	0.994	1.011	0.988	0.947	0.995	1.046	1.038	1.037	0.991	1.030

Data from Boltzmann fits (insets to Fig 4B)

	control slope mV)	1 butanol slope (mv)	control V50 (mV)	1-butanol V50 (mV)
cell-1	7.7	10.2	1.0	-2.0
2	6.0	9.6	1.5	1.8
3	7.1	10.4	-0.2	-0.5
4	7.2	10.0	1.6	1.8
5	4.6	8.5	0.8	1.9
6	6.2	8.8	-1.1	-3.4

Figure 4C (tert-butanol data)

	control normalized tail amplitude						1-butanol normalized tail amplitude					
X axis (mV)	cell-1	2	3	4	5	6	cell-1	2	3	4	5	6
-40	0.034	0.004	0.017	0.015	0.026	0.014	0.066	0.012	0.014	0.025	0.031	0.033
-30	0.055	0.012	0.037	0.044	0.052	0.053	0.081	0.039	0.053	0.048	0.040	0.077
-20	0.014	0.038	0.136	0.050	0.126	0.081	0.203	0.223	0.187	0.194	0.215	0.294
-10	0.153	0.203	0.296	0.270	0.346	0.293	0.516	0.668	0.470	0.562	0.523	0.673
0	0.404	0.637	0.711	0.650	0.686	0.700	0.809	0.853	0.711	0.804	0.770	0.897
10	0.663	0.865	0.894	0.850	0.884	0.932	0.900	0.908	0.903	0.895	0.899	0.950
20	0.896	0.951	0.959	0.926	0.945	1.001	0.967	0.965	0.972	0.952	0.915	0.979
30	1.014	0.967	1.060	0.938	1.000	1.020	0.986	0.980	1.000	0.987	0.936	1.011
40	1.000	1.000	1.000	1.000	1.000	1.000	1.000	1.000	1.000	1.000	1.000	1.000
50	1.008	0.973	1.047	0.996	1.043	0.981	0.966	1.017	0.983	0.972	0.964	0.982

Data from Boltzmann fits (insets to Fig 4C)

	control slope mV)	tert-but slope (mv)	control V50 (mV)	tert-but V50 (mV)
cell-1	7.5	6.6	4.2	-10.0
2	5.5	5.9	-3.0	-13.4
3	7.0	8.0	-4.5	-8.6
4	6.5	7.0	-3.7	-11.5
5	7.6	7.5	-4.8	-11.1
6	5.5	6.0	-4.7	-14.1

Fig 5C: time constant from exponential fit of tail current decays (seconds)			
control	1-butanol	control	tert-butanol
2.61E-04	2.22E-04	2.50E-04	3.98E-04
3.38E-04	2.28E-04	4.43E-04	6.98E-04
2.95E-04	2.16E-04	2.58E-04	3.11E-04
2.67E-04	1.85E-04	3.28E-04	6.17E-04
3.15E-04	2.28E-04	4.28E-04	3.90E-04
3.24E-04	2.25E-04	3.44E-04	6.72E-04

Figure 6: Inactivation curves (normalized current amplitude Vs conditioning potential), and data extracted from Boltzmann fits

X axis conditioning potential (mV)	control normalized amplitude cell-1	2	3	4	5
-90	1.00	1.00	1.00	1.00	1.00
-80	1.01	0.98	0.98	1.00	1.02
-70	0.93	0.96	0.94	0.99	0.99
-60	0.78	0.92	0.81	0.98	0.89
-50	0.58	0.84	0.61	0.92	0.75
-40	0.39	0.68	0.42	0.84	0.59
-30	0.22	0.39	0.24	0.60	0.42
-20	0.10	0.09	0.09	0.28	0.22

1-butanol normalized amplitude cell-1	2	3	4	5
1.00	1.00	1.00	1.00	1.00
0.83	0.99	0.90	1.01	0.96
0.65	0.94	0.70	0.97	0.78
0.42	0.80	0.48	0.86	0.58
0.29	0.65	0.31	0.71	0.38
0.13	0.45	0.17	0.53	0.21
0.00	0.17	0.05	0.25	0.06
0.00	0.02	0.00	0.04	0.00

V50 values from Boltzmann fit (mV)	control	1-butanol
cell-1	-47.0	-65.8
2	-33.9	-44.1
3	-43.7	-63.0
4	-27.0	-40.4
5	-35.5	-58.1

X axis conditioning potential (mV)	control normalized amplitude cell-1	2	3	4	5	
-90	1	1	1	1	1	1
-80	0.99422	1.00665	1.01661	1.02708	0.99523	0.99467
-70	0.94013	0.96931	0.90876	1.01085	0.99208	0.97967
-60	0.80692	0.87935	0.88556	0.99668	0.94545	0.87089
-50	0.58273	0.68745	0.73909	0.89946	0.80502	0.6879
-40	0.40285	0.47859	0.53688	0.77429	0.61031	0.51852
-30	0.23787	0.3198	0.35194	0.60499	0.27253	0.34358
-20	0.09402	0.16634	0.19845	0.34646	0.07327	0.15111

tert-butanol normalized amplitude cell-1	2	3	4	5	
1	1	1	1	1	1
0.79745	0.80441	1.06081	1.01571	0.89758	0.93107
0.54395	0.59583	1.02988	0.94982	0.76645	0.77773
0.31389	0.43446	0.76595	0.88867	0.57903	0.57993
0.15989	0.28631	0.57137	0.70458	0.38916	0.40136
0.06491	0.15126	0.36615	0.55751	0.16852	0.18061
0.00896	0.03163	0.14975	0.3514	0.0199	0.02395
0	0	0	0.06022	0	0

V50 values from Boltzmann fit (mV)	control	tert-butanol
cell-1	-44.5	-67
2	-40	-63
3	-38	-48
4	-27	-39
5	-37	-57
6	-40	-56.5

From figure 7B
decay of current amplitude: R490 values (current amplitude at 490ms / peak amplitude)

control	1-butanol	control	tert-butanol
0.413	0.156	0.388	0.233
0.511	0.194	0.492	0.288
0.470	0.108	0.363	0.180
0.502	0.199	0.480	0.247
0.654	0.169	0.627	0.274
0.566	0.184	0.711	0.351
0.586	0.185	0.590	0.252
0.606	0.265		
0.594	0.071		

Figure 7C: % inhbition of current charge

1-butanol	tert-butanol
65.6	46.3
45.6	27.6
67.4	45.8
65.5	28.5
68.6	34.5
66.7	46.5
70.4	55.9
61.2	
75.3	
